# Identification of key residues of B cell epitopes in hemagglutinin of H6 influenza A virus

**DOI:** 10.1128/spectrum.02059-23

**Published:** 2023-10-26

**Authors:** Zhimin Wan, Jianxi Gong, Jianjun Sang, Wenjie Jiang, Zhehong Zhao, Ting Tang, Yafeng Li, Yichao Zhao, Qiuqi Kan, Quan Xie, Tuofan Li, Hongxia Shao, Wei Gao, Aijian Qin, Jianqiang Ye

**Affiliations:** 1 Key Laboratory of Jiangsu Preventive Veterinary Medicine, Key Laboratory for Avian Preventive Medicine, Ministry of Education, College of Veterinary Medicine, Yangzhou University, Yangzhou, Jiangsu, China; 2 Jiangsu Co-innovation Center for Prevention and Control of Important Animal Infectious Diseases and Zoonoses, Yangzhou, Jiangsu, China; 3 Joint International Research Laboratory of Agriculture and Agri-Product Safety, the Ministry of Education of China, Yangzhou University, Yangzhou, Jiangsu, China; 4 Institute of Agricultural Science and Technology Development, Yangzhou University, Yangzhou, Jiangsu Province, China; 5 Sinopharm Yangzhou VAC Biological Engineering Co. Ltd, Yangzhou, Jiangsu, China; Changchun Veterinary Research Institute, Changchun, China

**Keywords:** H6, monoclonal antibody, escape mutant, antigenic epitope, passitive treatment

## Abstract

H6 influenza A viruses are enzootic and genetically diverse in both domestic poultry and wild birds, occasionally causing spillovers to mammals, including humans, posing threat to public health. However, the antigenic sites of the hemagglutinin (HA) of H6 are poorly understood. In this study, a panel of murine monoclonal antibodies (MAbs) with different reactivity spectra against HA of H6 was generated and nine key residues of antigenic epitopes in H6 were identified through escape mutants of these MAbs. Of these, residues 139 and 140 belonged to antigenic Sa, residue 89 belonged to antigenic Cb, residue 149 belonged to Ca2, while residue 221 belonged to Ca1 regarding the H1 antigenic sites. However, the other four residues 69, 120, 124, and 246 were not located in the identified antigenic regions of H1. Although most HA variants of the escape mutants had the preference for avian-like receptor, whereas escape mutants m3B9 and m4C2 increased the binding affinity for both avian and human-like receptors, and m1A5 and m6D11 decreased the binding affinity for avian-like receptors. Moreover, a single dose of MAbs 4C2 or 6E3 could fully protect mice against lethal challenge of mouse-adapted A/Eurasian teal/Jiangxi/2018WB0417(H6N2) (MA E-Teal/417) in the prophylactic treatment and also could provide partial protection in the therapeutic treatment. Overall, the generated MAbs and the identified key residues of antigenic epitopes not only provide novel insights into the key markers for monitoring the antigenic variation of H6 but also demonstrate the potential for developing MAb-based passitive treatments for H6.

**IMPORTANCE:**

Since the escape immunity of influenza A viruses (IAVs) is mainly caused by the continuous antigenic variations in HA, the identification of key antigenic epitopes is crucial for better understanding of the escape immunity and vaccine development for IAVs. The antigenic sites of several HA subtypes, including H1, H3, H5, and H9, have been well characterized, whereas those of H6 subtype are poorly understood. Here, we mapped nine key residues of antigenic epitopes in H6 through escape mutants using a panel of MAbs. Moreover, MAbs 4C2 and 6E3, targeting 140 and 89 residues, respectively, could protect mice against lethal challenge of MA E-Teal/417. These key residues of antigenic epitopes identified here provide the molecular targets for further elucidating the antigenic evolution of H6 and better preparing the vaccine against H6 IAV.

## INTRODUCTION

H6 subtype influenza A virus (IAV) was first isolated from turkeys in 1965 (1). Currently, H6 has become one of the most predominant subtypes of IAVs circulating in wild birds and domestic poultry globally (2 – 4) and is undergoing constant reassortant with different subtypes of IAVs (5 – 8). H6 IAVs are frequently introduced into poultry from wild birds; however, most of these introductions only resulted in limited transmission in poultry. From 2002 to 2005, H6N2 IAVs caused outbreaks and became enzootic among domestic poultry in California, USA (9). In southern China, H6 is the predominant subtype in live bird markets (10 – 12). Notably, H6 can cross-species and adapt to mammalians, posing a significant threat to public health. A certain proportion of H6 IAVs circulating in poultry in China can bind human-like receptor (11, 13), and H6N6 has been isolated from pigs in China since 2010 (14, 15). In 2013, a case of human infection with H6N1 virus was reported in Taiwan (16). In addition, several mutations in H6 viruses have been identified as virulence factors in mice (17, 18).

Hemagglutinin (HA) is the most abundant glycoprotein on the surface of IAV, which mediates binding to sialic acid on the host cells and facilitates fusion between the viral envelope and the host cell membrane (19). Due to the exposure on the viral surface and the biological functions, HA is the primary antigenic target of neutralizing antibodies. Antibodies targeted the globular head of HA are immunodominant, affinity-matured, bind with high specificity, and are generally neutralizing by interfering with the binding of HA to the sialic acids (20). Therefore, the globular head of HA is under the greatest immune pressure, resulting in continuous antigenic drift. HA immunodominance patterns of several HA subtypes have been characterized. Five major antigenic sites surrounding the receptor site Sa, Sb, Ca1, Ca2, and Cb in H1N1, site A, site B, site C, site D, and site E in the head domain in H3N2 and four major antigenic sites H9-A, H9-B, site I, and site II in H9N2 were identified, respectively (21 – 24). Since differences in the sequence and structure of HAs contribute to the particular patterns of immunodominance in animal models or humans, it is necessary to identify the antigenic sites of other HA subtypes, such as H6 IAV.

In the present study, we generated and characterized a panel of murine monoclonal antibodies (MAbs) against the HA of a mouse-adapted H6N2 strain, which was isolated from wild bird and adapted in BALB/c mice (18, 25). Moreover, we mapped HA key residues of antigenic epitopes by selecting escape mutants for these MAbs and tested the protection of MAbs 4C2 and 6E3 against mouse-adapted A/Eurasian teal/Jiangxi/2018WB0417(H6N2) (MA E-Teal/417) challenge in a mouse model.

## RESULTS

### Generation and characterization of MAbs specific to HA of H6 virus

**Fig 1:**
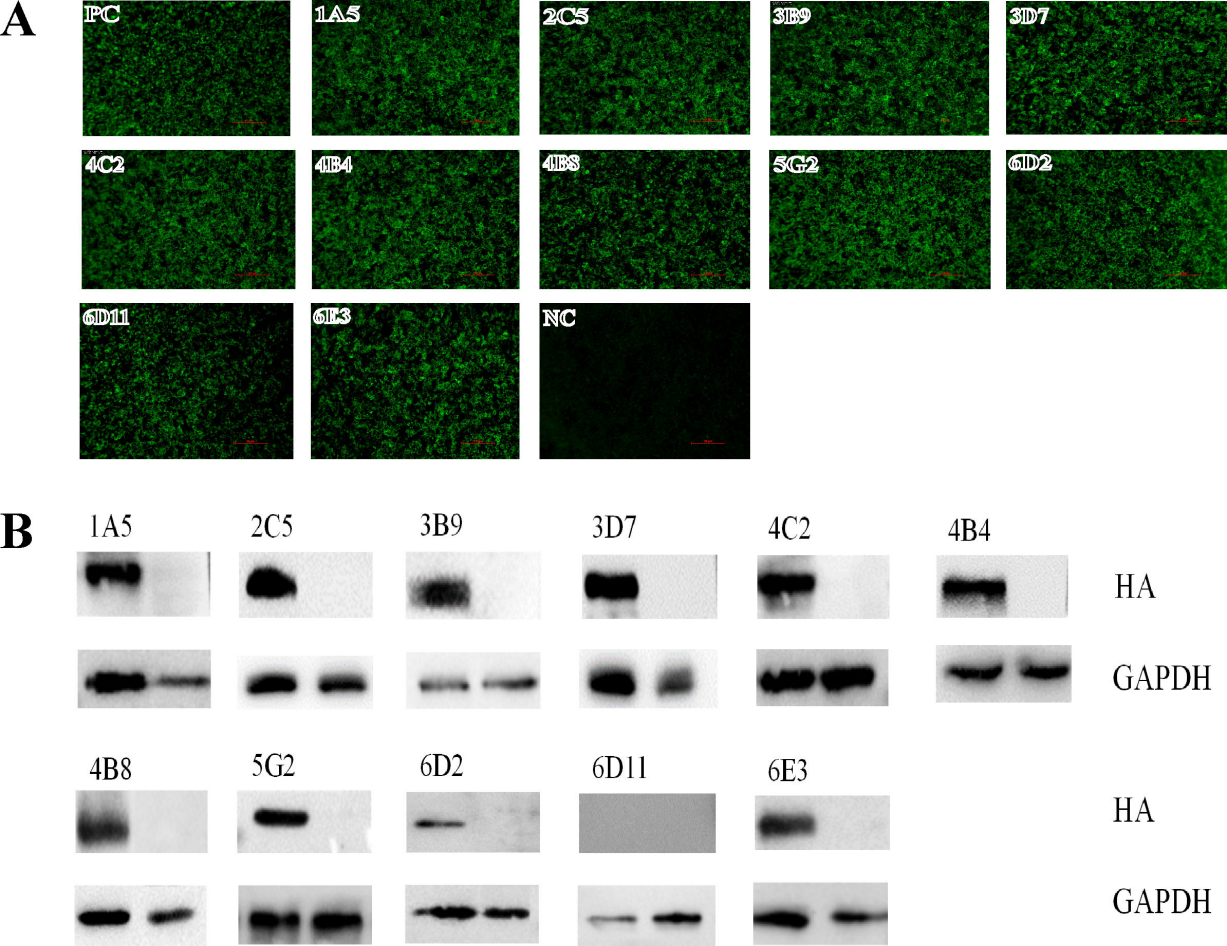
Characterization analysis of the 11 MAbs by IFA and Western blot assay. (**A**) Immunofluorescence and (**B**) Western blot of MAbs binding to HA of MA E-Teal/417. 293T cells were transfected with pDP2002-HA (MA E-Teal/417) and stained with each MAb.

**Fig 2:**
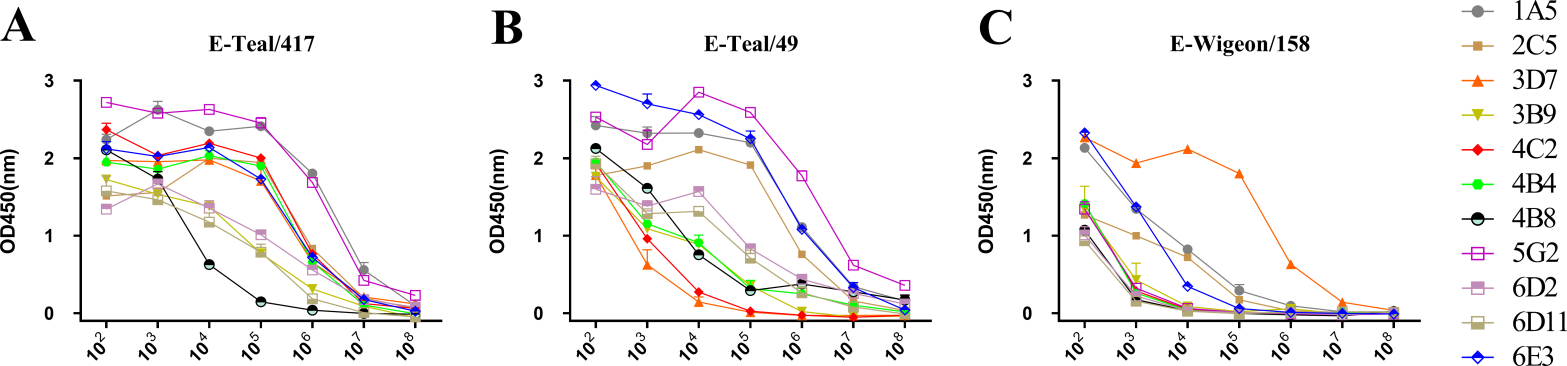
The binding ability of the 12 MAbs against the different lineages of H6 viruses. Binding of MAbs to (**A**) E-Teal/417, (**B**) E-Teal/49, and (**C**) E-Wigeon/158 in ELISA.

Through the fusion of mouse myeloma Sp2/0 cells with splenocytes from a mouse immunized with MA E-Teal/417 virus, a total of 457 murine hybridomas were screened, of which 25 hybridomas secreted antibodies against HA of E-Teal/417. Eleven murine hybridomas, secreting MAbs specific H6, were finally generated by two rounds of suncloning, named as 1A5, 2C5, 3B9, 3D7, 4C2, 4B4, 4B8, 5G2, 6D2, 6D11, and 6E3. Isotype detection showed that the isotype of 1A5, 4B4, 4B8, 4C2, 5G2, and 6E3 was IgG2b, the isotype of 3B9 and 6D11 was IgG1, and that of 2C5, 3D7, and 6D2 was IgG2a (Table 1). The specificity of these MAbs for H6 was confirmed by immunofluorescence assay (IFA). These MAbs recognized the HA protein expressed in 293T cells transfected with pDP2002-HA (MA E-Teal/417), which contains the HA gene of MA E-Teal/417 in pDP2002 plasmid (Fig. 1A). To further confirm the specificity of these MAbs, we tested these MAbs by hemagglutination inhibition (HI) assay, Western blot assay, and ELISA. In the HI assays, all these MAbs could inhibit MA E-Teal/417, with HI titers of 1:64–1:2,048 (Table 1). In the Western blot assays, all MAbs except for 6D11 could efficiently recognize the HA protein expressed in 293T cells transfected with pDP2002-HA (MA E-Teal/417) (Fig. 1B). In ELISA, all MAbs showed stronger binding activity to MA E-Teal/417 (Fig. 2A). To characterize the binding breadth of these H6-specific MAbs, we tested the binding activity of these MAbs with two other H6N2 viruses, A/Eurasian teal/Jiangxi/2018WB0049/2018(H6N2) (E-Teal/49, belonged to HN473-like), and A/Eurasian wigeon/Jiangxi/2018 WB0158/2018(H6N2) (E-Wigeon/158, belonged to ST2853-like), which were different genotypes from E-Teal/417 (25). The results showed that all MAbs could bind to these two H6 viruses; however, the MAbs had the different binding ability. As described in Fig. 2, all MAbs had the similar binding ability to E-Teal/417; however, MAbs 3D7, 6E3, and 1A5 had relatively strong binding ability to E-Wigeon/158, and other MAbs had lower binding ability to E-Wigeon/158 in ELISA (Fig. 2B and C). Compared with E-Teal/417, all MAbs reduced HI titers with E-Teal/49 and E-Wigeon/158 in HI assay, especially E-Wigeon/158 (Table 1). To further confirm the characteristics of the MAbs, these MAbs were purified and titrated with microneutralization assays against MA E-Teal/417 *in vitro*. All these MAbs showed neutralizing ability against MA E-Teal/417, and the range of 50% maximal inhibitory concentration (IC_50_) was from 0.37 to 76.8 μg/mL (Table 1).

**TABLE 1:** Biological properties of H6-specific MAbs in this study

MAbs^*a*^	Isotypes	HI titer			IC_50_ (μg/mL)
		E-Teal/417	E-Teal/49	E-Wigeon/158	E-Teal/417
1 A5	IgG2b	1:1024	1:256	1:64	76.8
2 C5	IgG2a	1:8192	1:2048	1:2048	0.8
3B9	IgG1	1:1024	1:512	1:16	0.4
3D7	IgG2a	1:256	1:64	1:128	4.8
4 C2	IgG2b	1:8192	1:512	1:32	0.4
4B4	IgG2b	1:4096	1:1024	1:32	0.39
4B8	IgG2b	1:32	1:8	1:4	12.5
5 G2	IgG2b	1:2048	1:2048	1:64	0.37
6D2	IgG2a	1:2048	1:512	1:32	0.6
6D11	IgG1	1:64	1:64	1:32	48.46
6E3	IgG2b	1:256	1:256	1:64	76.8

^*a*^ Untreated and purified mouse ascitic fluid of each hybridoma was used in HI assay and in IC50 titration, respectively.

### Selection of escape mutants and identification of key residues of B cell epitopes in HA of H6

**Fig 3:**
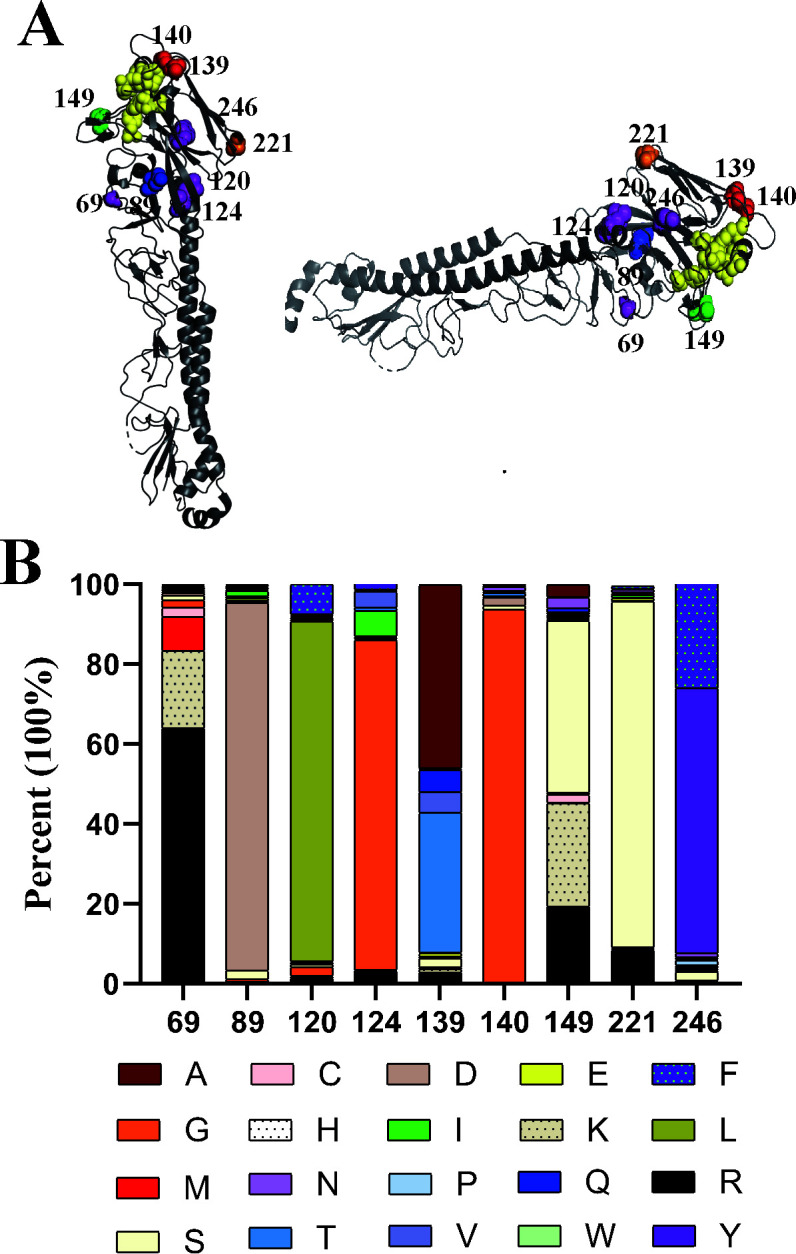
Location and natural mutations of nine critical amino acid positions in H6 antigenic sites. (**A**) The nine positions were identified by MAb escape mutants of MA E-Teal/417. The images were generated with Pymol software. Shown are the locations of these nine positions on H6 monomer (PDB:4WSS). The residues at 89 (Cb), 139 and 140 (Sa), 149 (Ca2), 221 (Ca1), 69, 120, 124, and 246 (overlap) were marked with blue, red, green orange, and purple, respectively. The conserved and variable residues (Y98, S136, W153, T155, N183, P186, V190, L194, L226, and G228) (H3 numbering) involving in receptor binding are colored yellow. (**B**) Natural mutations at nine key amino acid positions in the HA of H6 viruses. A total of 2,070 HA sequences of H6 viruses were downloaded from Influenza Research Database and were analyzed.

To localize the key residues of B cell epitopes targeted by these MAbs, escape mutants of MA E-Teal/417 were selected by co-culturing the virus and MAb in embryonated chicken eggs. The escape mutants were further purified by limiting dilution in eggs. A total of 15 escape mutants were generated by these 11 MAbs (Table 2). As expected, all mutant viruses were not inhibited by the corresponding selected MAbs in HI assay, except for m2C5, which had low a HI titer (1:4) with MAb 2C5 (Table 2). Among the 13 escape mutants, selected with MAbs 1A5, 2C5, 3B9, 3D7, 4B4, 5G2, 6D2, 6D11, and 6E3, respectively, each had a single amino acid mutation in its HA, at position 69, 89, 139, 140, and 149, respectively (Table 2). The mutant of m6E3 (selected with 6E3) had double mutations in HA, R69I, and S221N. The remaining one mutant m4B8 had three mutations in HA, L120F, R124G, and Y246H (Table 2). Taken together, we identified nine critical residues of antigenic epitopes in H6. As shown in Fig. 3A, these nine positions on H6 identified in this study were all located in the HA globular head region.

**TABLE 2:** Amino acid mutations in the HA of escape mutants selected with H6-specific MAbs

MAbs	Mutants	HI titer	Mutation (S)
1A5	m1A5	*–^a^*	R69G^*b*^
2C5	m2C5	1:4	K149E
3B9	m3B9-1	–	T139I
	m3B9-2	–	T139I
3D7	m3D7	–	D89N
4C2	m4C2	–	G140E
4B4	m4B4-1	–	G140E
	m4B4-2	–	G140E
4B8	m4B8	–	L120F, R124G, Y246H
5G2	m5G2	–	G140E
6D2	m6D2-1	–	G140E
	m6D2-2	–	G140R
6D11	m6D11	–	R69I, S221N
6E3	m6E3-1	–	D89N
	m6E3-1	–	D89N

^*a*^ Shown are the titers obtained with each mutant selected with MAb; –, no inhibition in HI assay.

^*b*^ H6 numbering.

### Cross-reactions of H6 escape mutants with H6-specific MAbs and anti-serum

To determine the effect of amino acid mutations in escape mutants on the antigenicity of H6 IAVs, the cross-reactions of these H6 escape mutants with the H6-specific MAbs and serum against MA E-Teal/417 were performed by HI assays. Due to the four mutants (m4C2, m4B4, m5G2, and m6D2), m3D7 and m6E3 had the mutation in the same positions (140 and 89), m4C2 and m6E3 were chosen to do the cross-reactions, and MA E-Teal/417 and anti-serum as control. As shown in Table 3, each escape mutant was inhibited by serum against MA E-Teal/417 and most if not all of the other MAbs at titers close to those of the selecting MAbs. Due to the four MAbs of 4C2, 4B4, 5G2, and 6D2, 3D7 and 6E3 targeted in the same positions in HA, it is not surprising that these MAbs cannot efficiently inhibit corresponding escape mutants (m4C2 and m6E3). Interestingly, m3B9 (T139I) and m4C2 (G140E) had low cross-reactivity with the corresponding MAbs. In addition, m1A5 had lower HI titers with most MAbs, including 1A5, 2C5, 3B9, 4B4, 4C2, 6D2, and 6D11 (Table 3).

**TABLE 3:** Cross-reactions of H6 escape mutants with H6-specific MAbs and anti-serum

MAb	Inhibition of viruses^*a*^							
	E-Teal/417	m1A 5	m2C5	m3B9	m4B8	m4C2	m6D11	m6E3
Sera^*b*^	+	+	+	+	+	+	+	+
1A5	+	−	+	+	+	+	−	+
2C5	+	−	−	+	+	+	−	+
3B9	+	−	+	−	−	−	+	−
3D7	+	+	+	+	+	+	+	+
4B4	+	−	+	−	+	−	+	+
4B8	+	+	+	+	−	+	+	+
4C2	+	−	+	−	+	−	+	+
5G2	+	−	+	−	+	−	+	+
6D2	+	−	+	−	+	−	+	+
6D11	+	−	+	+	+	+	−	+
6E3	+	+	+	+	−	+	+	−
Neg ctrl^*c*^	−	−	−	−	−	−	−	−

^*a*^ +, HI titer ≤ eightfold different from that obtained with MA E-Teal/417; −, HI titer ≥16-fold different from that obtained with ma E-Teal/417, for negative control (Neg ctrl), no inhibition.

^*b*^ Sera, the mouse sera against ma E-Teal/417.

^*c*^ Neg ctrl, ascitic fluid containing H9-specical MAb 2G10.

### Natural mutations in the identified key residues of antigenic epitopes in H6 field strains

To examine whether these identified antigenic epitopes in H6 HA have undergone mutations in the field, we examined 2070 full-length HA sequences of H6 viruses deposited in Influenza Research Database (www.bv-brc.org) for variations at each of the nine amino acid positions. As described in Fig. 3B; Table 4, some of these positions, such as 69, 139, 149, and 246, were highly variable, while the amino acids of positions 89 and 140 were comparatively conserved, as evidenced by less than 10% variations among the 2,070 sequences analyzed, Interestingly, except the position 246, the other 8 mutations (R69G, D89N, L120F, R124G, T139I, G140E, G140R, and K149E) detected in the HA of the escape mutants in this study were already present in some of the natural H6 isolates.

**TABLE 4:** Natural mutations at nine key amino acid positions in the HA of H6 viruses

AA position^*a*^	Mutation (% of isolates bearing each residue)^*b*^														
69	R (64.0)	K (19.4)	M (8.5)	C (2.2)	G (1.9)	S (1.2)	D (0.6)	E (0.5)	L (0.4)	W (0.4)	I (0.4)	P (0.2)	T (0.2)	V (<0.1)	Q (<0.1)
89	D (92.1)	S (2.2)	I (1.3)	G (0.6)	L (0.6)	W (0.5)	E (0.5)	R (0.4)	N (0.4)	Q (0.3)	Y (0.3)	V (0.2)	A (0.2)	C (0.1)	P (0.1)
	F (<0.1)	K (<0.1)													
120	L (85.0)	F (7.5)	G (2.2)	R (1.4)	S (0.7)	D (0.5)	K (0.48)	E (0.4)	T (0.4)	V (0.4)	Y (0.3)	A (0.2)	Q (0.2)	P (0.1)	C (<0.1)
	N (<0.1)	I (<0.1)													
124	G (82.4)	I (6.3)	V (4.0)	R (3.3)	F (1.4)	T (0.7)	E (0.4)	N (0.4)	W (0.3)	M (0.2)	D (0.2)	S (0.2)	A (0.1)	P (<0.1)	Y (<0.1)
	C (<0.1)														
139	A (45.9)	T (34.9)	Q (5.4)	V (5.2)	R (2.9)	S (2.2)	K (0.8)	E (0.7)	D (0.5)	G (0.5)	N (0.4)	P (0.2)	Y (0.1)	L (<0.1)	F (<0.1)
	I (<0.1)	H (<0.1)													
140	G (93.4)	D (2.0)	N (1.0)	S (1.0)	T (0.8)	Y (0.4)	V (0.4)	A (0.3)	L (0.2)	E (0.2)	Q (0.2)	R (0.1)	K (<0.1)	F (<0.1)	I (<0.1)
149	S (42.9)	K (25.8)	R (19.4)	A (3.0)	N (2.6)	C (2.0)	Q (1.0)	G (0.6)	P (0.6)	V (0.5)	E (0.4)	I (0.3)	L (0.3)	T (0.2)	Y (0.2)
	F (<0.1)	M (<0.1)													
221	S (86.4)	R (8.4)	I (0.7)	N (0.7)	E (0.7)	F (0.7)	G (0.4)	P (0.4)	K (0.3)	D (0.2)	L (0.2)	T (0.2)	M (0.1)	A (<0.1)	Y (<0.1)
246	Y (66.4)	F (26.0)	S (2.2)	P (1.2)	N (1.0)	W (0.6)	L (0.5)	V (0.5)	E (0.4)	K (0.4)	G (0.3)	T (0.3)	A (<0.1)	C (<0.1)	D (<0.1)
	I (<0.1)	M (<0.1)													

^*a*^ AA, amino acid; AA position, H6 numbering.

^*b*^ A total of 2,070 full-length H6 sequences available in Influenza Research Database.

### Effect of escape mutants on viral receptor-binding preference

**Fig 4:**
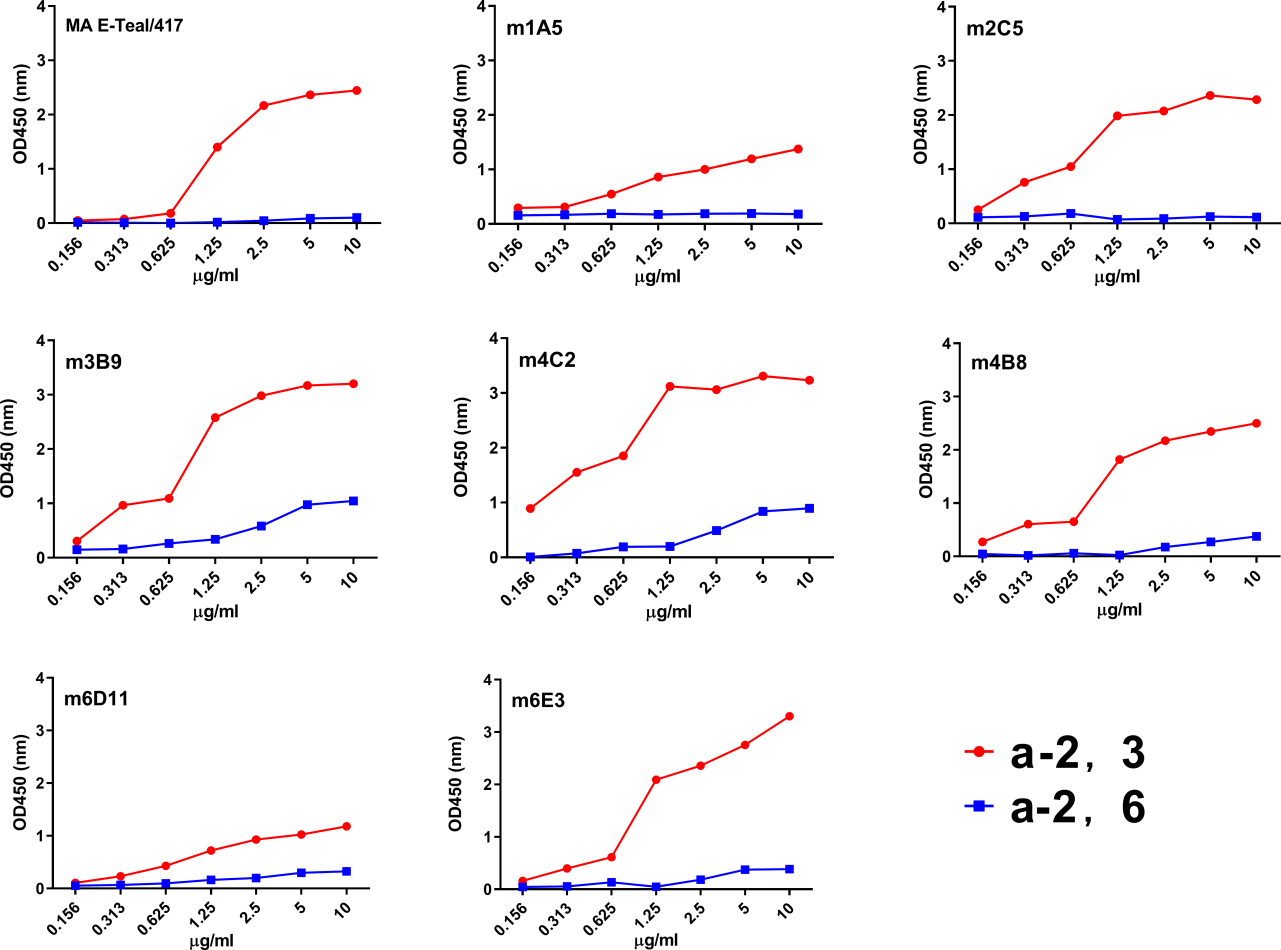
Receptor-binding properties of MA E-Teal/417 and escape mutants. The binding of the viruses with sialic acids was determined using various concentrations of sialic acids conjugated to biotinylated sialylglycopolymers (3′SLN and 6′SLN) via direct solid-phase binding assays.

Previous studies have shown that MA E-Teal/417 mainly binds to α-2.3 sialic acid (SA) (18). To determine whether amino acid substitutions of the escape mutants alter the viral receptor-binding preference, the escape mutants, m1A5 (targeting residue 69), m2C5 (targeting residue 149), m3B9 (targeting residue 139), m4C2 (targeting residue 140), m4B8 (targeting residues 120, 124, and 246), m611 (targeting residues 69 and 221), m6E3 (targeting residue 89), were tested with two receptor analogs, i.e., Neu5Acα2–3Galb1-4GlcNAcb (3′SLN) and Neu5Acα2–6Galb 1–4GlcNAcb (6′SLN), for receptor-binding preference. As described in Fig. 4, the escape mutants m1A5, m2C5, m4B8, m6D11, and m6E3 maintained the same receptor-binding properties as MA E-Teal/417 virus; however, the escape mutants m3B9 and m4C2 increased the binding affinity for both α-2.3 SA and α-2.6 SA, and m1A5 and m6D11 decreased the binding affinity for α-2.3 SA.

### Effective treatment of MAbs of 4C2 and 6E3 is against H6 virus in mice

**Fig 5:**
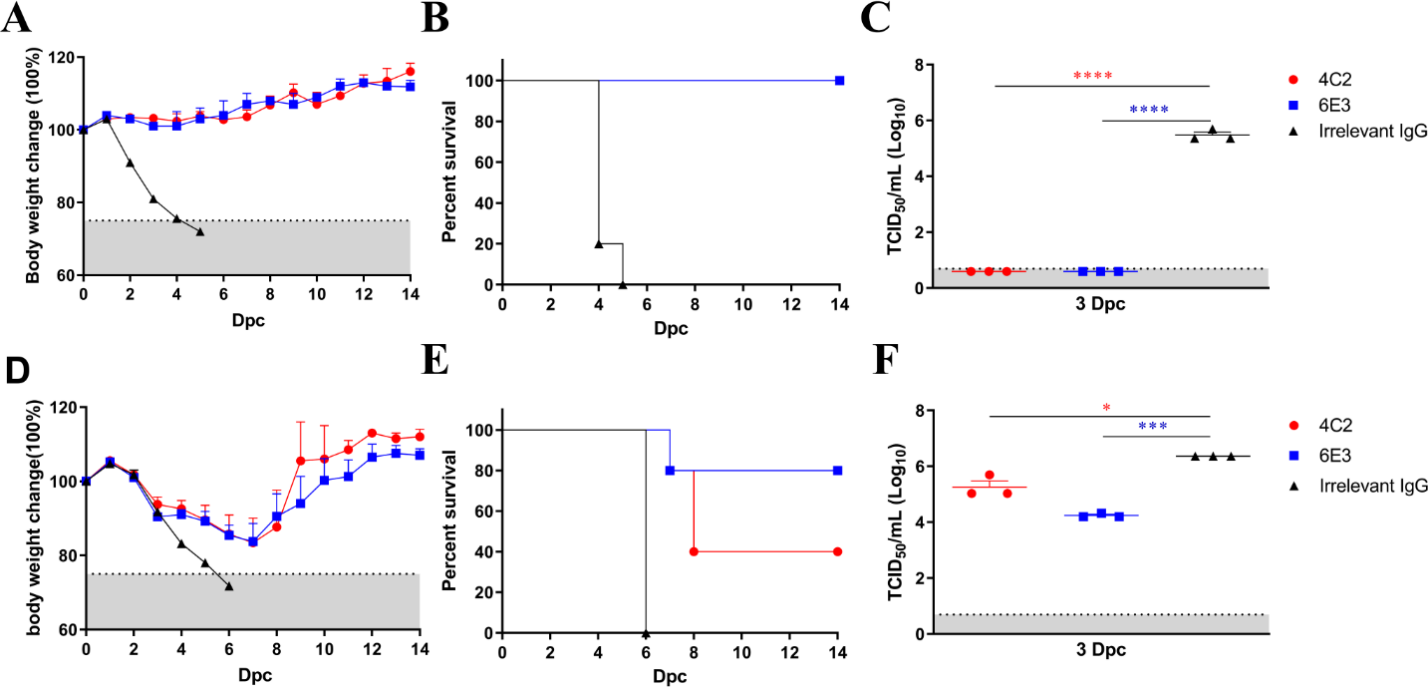
Prophylactic and therapeutic efficacy of MAbs in mice against MA T-Teal/417 challenge. Five-week-old BALB/c mice (8/group) were treated with MAb 4C2 or 6E3 (15 mg/kg) intraperitoneally 12 h before (**A through C**) or 12 h after (**D through F**) challenge with 10 MLD_50_ of MA E-Teal/417. Mice treated with H9-special MAb 2G10 served as a control. Body weight change (**A and D**) and mortality (**B and E**) were monitored daily for 14 days, and on 3 dpc, three mice in each group were euthanized, and lung viral titers (**C and F**) were determined in MDCK cells. The statistical analysis was performed with a Student’s *t-*test. A *P* value of below 0.05 was considered significant. *, **, ***, and **** indicate *P* values of less than 0.05, 0.01, 0.001, and 0.0001, respectively.

Since MAbs 4C2 and 6E3 target amino acids in the HA positions of 140 and 89, which are comparatively conserved in H6 viruses, we evaluated their ability to protect mice against lethal challenge with MA E-Teal/417. In the prophylactic experiments, MAbs 4C2 and 6E3 were administered intraperitoneal injection (i.p.) to two groups of mice (*n* = 8/group) 12 h before 10 50% mouse lethal dose (MLD_50_) of MA E-Teal/417 challenge. As shown in Fig. 5A, a single dose of 4C2 or 6E3 at 15 mg/kg fully protected all mice from the MA E-Teal/417 challenge, and mice treated with 4C2 and 6E3 did not lose any body weight after challenge. In contrast, the body weights of mice in the control group declined rapidly, and by 5-day-post challenge (dpc), all mice had succumbed to the challenge (Fig. 5A and B). Three mice of each group were euthanized, and the lung samples were collected to determine the viral load at 3 dpc, respectively. Mice of the control group showed high viral titers in the lungs, and mice treated with 4C2 or 6E3 had no detected viral titers in the lungs (Fig. 5C).

In the therapeutic experiments, MAbs 4C2 or 6E3 were administered to mice 12 h after 10 MLD_50_ of MA E-Teal/417 with the same way and same dose as above. Three mice of each group were euthanized, and the lungs were collected to determine the viral load at 3 dpc. The results showed that the body weight of mice treated with 4C2 or 6E3 declined from 2 dpc and the mice recovered from 8 dpc (Fig. 5D). Eighty percent (4/5) and 40% (2/5) of mice treated with 6E3 and 4C2, respectively, survived the challenge by 14 dpc (Fig. 5E). In contrast, the body weights of mice in the control group declined rapidly, and all mice died or had to be euthanized at 6 dpc (Fig. 5E). For the virus load in the lungs, as shown in Fig. 5F, the viral titers in mouse lungs of 4C2 and 6E3 groups were significantly reduced (about 10- and 100-fold, respectively) compared with the control group (Fig. 5F).

**Fig 6:**
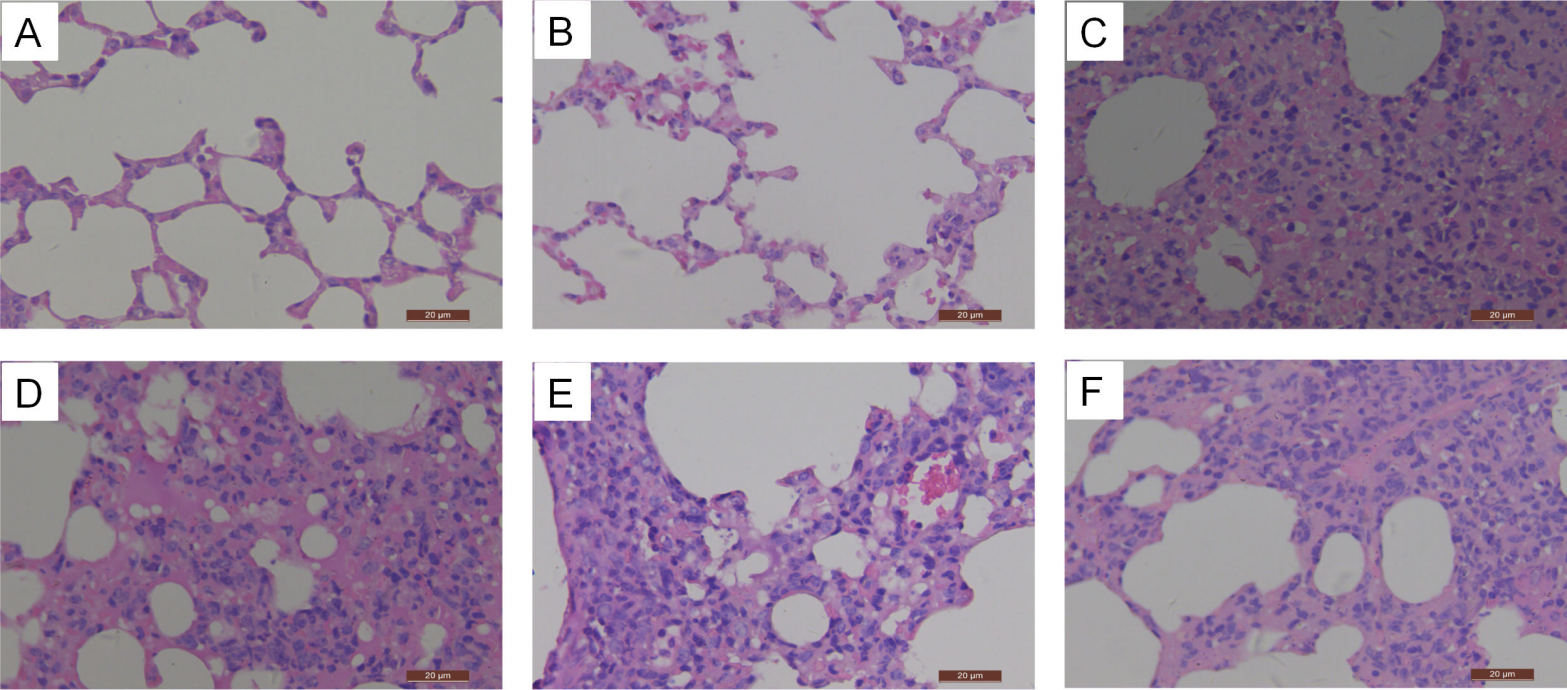
Histological analysis of mouse lungs challenged with MA-Teal/417. The lungs collected at 3 dpc were fixed in 10% formalin, embedded in paraffin, and sectioned. Serial section was stained with H&E. The lungs of prophylactically treated with 4C2 (**A**), 6E3 (**B**), and irrelevant antibody (**C**) and therapeutically treated with 4C2 (**D**), 6E3 (**E**), and irrelevant antibody (**F**) were analyzed.

To further evaluate the protective efficacy of 4C2 and 6E3, the lungs of each group were histopathologically analyzed. In both the prophylactic and therapeutic experiments, the lung tissues of mice in the control group exhibited interstitial pneumonia with congestion, and the alveolar wall was significantly thinned (Fig. 6C and F). Compared with the control group, the histopathological lesions in the lung tissues of mice treated with MAb 4C2 or 6E3 were attenuated. The lesions of lung tissues of mice prophylactically treated with 4C2 or 6E3 were barely visible (Fig. 6A and B). In the therapeutic experiments, the lung tissues in mice treated with 4C2 exhibited focal interstitial pneumonia with diffuse inflammatory cells around the bronchus, accompanied by edema, and the lung tissues in mice treated with 6E3 also showed mild inflammation (Fig. 6D and E).

## DISCUSSION

Substitutions in key antigenic sites in the HA and NA proteins of influenza viruses greatly affect viral antigenic properties, which generally causes viruses to acquire the ability to escape natural or vaccine-induced immunity and lead to vaccine failure in the field. This failure can result in the virus circulating and spreading unhindered in the vaccinated animal, including humans (26, 27). Therefore, the identication of the vital antigenic sites in HA or NA is critical for better preparing the effective vaccine or super-antigen with cross-protective potentials. Although several HA antigenic domains of IAVs, such as H1, H3, and H9, have been characterized (21 – 23), those of other HA subtypes need to be further identified.

In this study, 11 murine H6-specific MAbs were generated and used to identify H6 key residues of antigenic epitopes by selecting escape mutants against MAbs. A total of 15 escape mutants were generated by these 11 MAbs, and 9 critical residues of antigenic epitopes in H6 were identified. Due to both H6 and H1 belonging to group 1 of influenza A virus and antigenic sites of H1N1 have been classified as the sites Sa, Sb, Ca1, Ca2, and Cb (21, 22, 28), we divided these nine critical residues of antigenic epitopes identified in H6 based on the classification of H1 antigenic sites. These at position 139 targeted by MAb 3B9, and 140 targeted by MAb 4C2, 4B4, 5G2, and 6D2 are comparable to those in antigenic Sa, and residue 149 targeted by MAb 2C5 belongs to Ca2, 89 targeted by MAbs 3D7 and 6E3 belongs to Cb, the position 221 belonged to Ca1, and the other four positions of 69, 120, 124, and 246, are located out of the H1 antigenic site. All these demonstrate that 5 MAbs (45.5%) targeted Sa, 2 MAbs (18.2%) targeted Cb, and 2 MAbs target to Ca1 and Ca2, respectively. In addition, the MAbs targeting antigenic sites Sa (positions 139 and 140) showed stronger neutralizing activity than other MAbs (Table 1). These data suggest that Sa is the immunodominant in H6 virus and can induce strong neutralizing antibodies. Among these H6-specific MAbs, some MAbs, such as 1A5, 4C2, and 6E3, have a broad reactivity spectrum (Fig. 2) and target conserved antigen residues (Table 4; Fig. 3); however, the antigenic residues targeted by the other MAbs were not conserved. The characteristics of these MAbs and the reactivity spectra of H6 strains with these MAbs can be used to monitor the evolution of HA antigenicity of H6 IAVs.

The virus MA E-Teal/417 used for selection of escape mutants was generated through adaptation of E-Teal/417 in BALB/c mice (18). Compared with wild-type E-Teal/417, MA E-Teal/417 has the G124R mutation in HA (18). Interestingly, the position 124 is targeted by MAb 4B8, residue 124 of m4B8 returns to G in HA, indicating that the residue 124 of E-Teal/417 is not only a key residue of antigenic epitope but also a virulence marker. Whether the pathogenicity of m4B8 is decreased needs to be further investigated. Receptor binding is an important determinant of host specificity, and modification of receptor-binding property is a critical step for cross-species transmission. Some H6 strains can bind to human-like receptors (11, 13). In this study, we found that the escape mutants m3B9 (T139I) and m4C2 (G140E) increased the binding affinity for both avian and human-like receptors, and m1A5 and m6D11 decreased the binding affinity for avian-like receptors (Fig. 4). Residues 139 and 140 belong to Sa regarding the H1 antigenic sites. As shown in Fig. 4A, these two residues were close to receptor-binding area in H6 structure, which suggest that these two amino acid mutations might cause the conformational change of the receptor binding site of H6. Notably, the mutations of T139I and G140E are already present in some of the field H6 isolates, highlighting that the continued surveillance of H6 viruses for such mutations is critical in the field.

Currently, vaccination is the major strategy to control influenza virus infection; however, influenza vaccines generally induce narrow, strain-specific immune responses, and their effectiveness varies depending on how well the vaccines match the circulating strains. Moreover, the available vaccines also do not efficiently protect against new pandemics or emerging viruses. Therefore, antiviral treatment needs to be developed against pandemic or emerging influenza viruses. H6 subtype of IAVs has a wide range of hosts, including birds, swine, and humans, which may become the next pandemic. However, there is no H6 vaccine available, so it is important to develop antivirus drugs. Therapeutic MAb drugs can be as important candidates for fighting the infectious diseases, which have been approved for clinical treatment of respiratory syncytial virus, Ebola virus, and SARS-CoV-2 (29 – 31). In this study, MAbs 4C2 and 6E3 were selected for protective treatment in a mouse model. Our data showed that a single dose of two MAbs could fully protect mice against lethal challenge in the prophylactic group, and these two MAbs could effectively reduce virus replication in the lungs in the therapeutic group (Fig. 5), highlighting the application of these two MAbs as therapeutic drug candidates in the potential pandemic of H6 in the future.

In summary, this is the first demonstration of the identification of nine novel residues of antigenic epitopes in H6 through escape mutants against MAbs. Moreover, two MAbs 4C2 and 6E3 can provide effective protection against H6 lethal challenge in a mouse model. These findings provide molecular markers for preparing efficient vaccines against H6, and potential antiviral drugs for fighting the infection of H6 in the future.

## MATERIALS AND METHODS

### Cells, viruses, and plasmids

Human 293T cells were maintained in Dulbecco’s modified Eagle’s medium (DMEM), containing 10% fetal calf serum (Lonsera, Shanghai, China) and 1% antibiotic. Mouse myeloma Sp2/0 and hybridoma cells were maintained in DMEM, containing 10% fetal calf serum, hypoxanthine, and thymidine (HT, Sigma-Aldrich) and 1% antibiotic. E-Teal/49, E-Wigeon/158, and E-Teal/417 were isolated from wild birds in Poyang Lake in China (25), and MA E-Teal/417 was generated by passaging in BALB/c mice (18). The viruses were amplified in 10-day-old specific-pathogen-free (SPF) embryonated chicken eggs and stored at −80°C. The pDP2002-HA (MA E-Teal/417) was constructed with the Mut Express II Fast Mutagenesis Kit V2 (Vazyme Biotech Co., Ltd).

### Preparation of murine MAbs

The MAbs used in this study were prepared as previously described (32). In brief, 6-week-old BALB/c mice were infected intranasally with MA E-Teal/417 virus and were immunized by i.p. of allantoic fluid containing MA E-Teal/417 at 3 and 6 weeks thereafter. Three days before the fusion, mice were boosted with MA E-Teal/417 virus intraperitoneally. Splenocytes from the immunized mouse were fused with mouse myeloma Sp2/0 cells. Hybridomas were screened with IFA. The isotype of each MAb was determined with the rapid ELISA mouse MAb isotyping kit. Ascitic fluid of each hybridoma was prepared in mice.

### IFA

IFA was performed following the previous protocol (33). Briefly, 293T cells were transfected with pDP2002-HA (MA E-Teal/417) in 96 well plates and fixed with cold acetone-alcohol (3:2, vol/vol). The plates were incubated with supernatant or ascitic fluid of hybridomas at 37°C for 30 min. After washed three times with PBS, the plates were incubated with goat anti-mouse IgG conjugated with fluorescein isothiocyanate (Sigma-Aldrich, USA). After washed three times with PBS, the cells were observed.

### HI assay

HI assay was performed to titrate HI titers of all MAbs. Briefly, MAbs were subsequently serially twofold diluted and mixed with eight hemagglutination units (HAUs) of virus in 96-well plates and incubated at room temperature (RT) for 30 min. The HI activity was visualized by adding 0.5% chicken red blood cells to the virus-MAb mixture and incubated at RT for 30 min before reading.

### ELISA

H6 viruses were coated overnight at 4°C in 96-well plates (64 HAUs/well). Then, the plates were blocked with 5% skim milk in PBS at 37°C for 2 h and then washed three times with PBS containing 0.5% Tween 20 (PBST). The plates were incubated with serially twofold diluted mouse ascitic fluid of each MAb at 37°C for 1 h. After washing three times with PBST, the plates were incubated with horseradish peroxidase (HRP)-conjugated IgG antibody at 37°C for 1 h. After washing five times with PBST, 100 µL of TMB solution was added to each well and incubated for 15 min at RT. The reaction was stopped by adding 2M H_2_SO_4_, and OD_450_ values were measured.

### Western blot assay

293T cells were transfected with pDP2002-HA (MA E-Teal/417) for 48 h. Then, the cells were harvested and lysed in lysis buffer with a mixture of proteolytic protease and phosphatase inhibitor (NCM, Soochow, China). The lysates were boiled in the loading buffer and then immediately subjected to 10% SDS-PAGE and transferred to nitrocellulose (NC) membranes (GE Healthcare life Sciences, Freiburg, Germany). After blocked with NcmBlot blocking buffer (NCM, Soochow, China), the membrane was incubated with each MAb for 1 h at RT. After washing three times with PBST, the membrane was incubated with HRP-conjugated IgG antibody for 1 h at RT. After washing three times with PBST, the membranes were developed with chemiluminescent reagents and imaged with an automatic imaging system (Tanon 5200).

### Titration IC_50_ of the MAbs

Each MAb was subsequently serially twofold diluted and mixed with MA E-Teal/417 (100 TCID_50_) and incubated for 30 min at RT. The mixture was then added to MDCK cells in 96-well plates and incubated for 2 h at RT, each concentration of MAb having four replicates. Then, the mixture was removed and added the fresh opti-MEM containing 1 µg/mL of TPCK-Trypsin. At 72 h post-infection, the supernatant were titrated by HA assays and IC_50_ was determined by four-parameter logistic regression.

### Selection of MAb escape mutants

Selection of MAb escape mutants was carried out as reported (34). MA E-Teal/417 was incubated with excess of mouse ascetic fluid containing MAb at 37°C for 30 min, the mixture was incubated into 10-day-old SPF embryonated chicken eggs. The presence of mutant was confirmed with HI assay and cloned by limiting dilution in 10-day-old SPF embryonated chicken eggs.

### PCR amplification of HA gene and sequence

Viral RNA was extracted from allantoic fluid containing virus with RNA Mini Kit (Vazyme Biotech Co., Ltd). Reverse transcription and PCR for amplification of HA gene were carried out as described (35). PCR products were sequenced. The sequence data were examined for nucleotide and amino acid mutations with DNAStar software.

### Solid-phase binding assay

The receptor-binding property of MA E-Teal/417 and the escape mutants were analyzed by solid-phase binding assay as previously described (18). Briefly, the Perce streptavidin high binding capacity coated 96-well plates (Thermo Fisher Scientific, USA) were coated serially twofold diluted Neu5Aca2-3Galb1-4GlcNAcb-PAA-biotin and Neu5Aca2-6Galb1-4GlcNAcb-PAA-biotin (Glycitech, USA) and incubated overnight at 4°C. After blocked with 5% skim milk in PBST, the plates were incubated at 4°C overnight with 64 HAUs of MA E-Teal/417 and escape mutants. After washed three times with PBST, the plates were incubated with serum containing antibodies against MA E-Teal/417. The plates were washed three times with PBST and incubated with horseradish peroxidase (HRP)-conjugated anti-mouse IgG antibody for 2 h at 4°C and then washed three times with PBST again. Then, 100 µL TMB solution was added to each well and incubated 10 min. The reaction was stopped by adding 2 M H2SO4, and the OD450 values were measured.

### Prophylactic and therapeutic studies

Five-week-old BALB/c mice were randomly assigned to experimental groups composed eight mice, five mice of which were used to detect bodyweight changes and survival, and three of which were used to titrate viral replication in the lungs. To examine the prophylactic efficacy of 4C2 and 6E3, which were targeted relatively conservative amino acids of H6 virus, MAbs were administered i.p. at 15 mg/kg of body weight 12 h before intranasal challenge with 10 MLD_50_ of MA E-Teal/417. On 3 dpc, three mice in each group were euthanized and the lungs were collected for virus titration in MDCK cells. To exam the therapeutic efficacy, MAbs were administered at the same dose (15 mg/kg) to mice 12 h after intranasal challenge with 10 LD_50_ of MA E-Teal/417. On 3 dpc, three mice in each group were euthanized and the lungs were collected for virus titration in MDCK cells. In all experiments, bodyweight and mortality were monitored for up to 14 days. Mice with body weight loss of more than 25% were humanely euthanized.

### Statistical analysis

The statistical analysis in this study was performed with a Student’s *t* test using GraphPad 8 software. A *P* value of below 0.05 was considered significant. *, **, ***, and **** indicate *P* values of less than 0.05, 0.01, 0.001, and 0.0001, respectively.

## References

[1] Downie JC , Webster RG , Schild GC , Dowdle WR , Laver WG . 1973. Characterization and ecology of a type A influenzavirus isolated from a sheawater. Bull World Health Organ 49:559–566. 4548383 PMC2481032

[2] Munster VJ , Baas C , Lexmond P , Waldenström J , Wallensten A , Fransson T , Rimmelzwaan GF , Beyer WEP , Schutten M , Olsen B , Osterhaus A , Fouchier RAM . 2007. Spatial, temporal, and species variation in prevalence of influenza A viruses in wild migratory birds. PLoS Pathog 3:e61. doi:10.1371/journal.ppat.0030061 17500589 PMC1876497

[3] Lee E-K , Kang H-M , Song B-M , Lee Y-N , Heo G-B , Lee H-S , Lee Y-J , Kim J-H . 2017. Surveillance of avian influenza viruses in South Korea between 2012 and 2014. Virol J 14:54. doi:10.1186/s12985-017-0711-y 28292308 PMC5351195

[4] Cardona C . 2004. “Low-pathogenicity avian influenza outbreaks in commercial poultry in California” The threat of pandemic influenza: are we ready? Workshop summary, Proceedings for the Symposium on pandemic influenza preparedness; Institute of Medicine

[5] Senne DA . 2003. Avian influenza in the Western Hemisphere including the Pacific Islands and Australia. Avian Dis 47:798–805. doi:10.1637/0005-2086-47.s3.798 14575067

[6] Woolcock PR , Suarez DL , Kuney D . 2003. Low-pathogenicity avian influenza virus (H6N2) in chickens in California, 2000-02. Avian Dis 47:872–881. doi:10.1637/0005-2086-47.s3.872 14575080

[7] Kim HR , Lee YJ , Lee KK , Oem JK , Kim SH , Lee MH , Lee OS , Park CK . 2010. Genetic relatedness of H6 subtype avian influenza viruses isolated from wild birds and domestic ducks in Korea and their pathogenicity in animals. J Gen Virol 91:208–219. doi:10.1099/vir.0.015800-0 19812266

[8] Araujo J , Petry MV , Fabrizio T , Walker D , Ometto T , Thomazelli LM , Scherer AL , Serafini PP , Neto IS , Krauss S , Webster RG , Webby RJ , Durigon EL . 2018. Migratory birds in Southern Brazil are a source of multiple avian influenza virus subtypes (vol 12, pg 220, 2018). Influenza Other Respir Viruses 12:220–231. doi:10.1111/irv.12519 29143465 PMC5820415

[9] Yee KS , Novick CA , Halvorson DA , Dao N , Carpenter TE , Cardona CJ . 2011. Prevalence of low pathogenicity avian influenza virus during 2005 in two U.S. live bird market systems. Avian Dis 55:236–242. doi:10.1637/9427-061610-Reg.1 21793439

[10] Chin PS , Hoffmann E , Webby R , Webster RG , Guan Y , Peiris M , Shortridge KF . 2002. Molecular evolution of H6 influenza viruses from poultry in Southeastern China: prevalence of H6N1 influenza viruses possessing seven A/Hong Kong/156/97 (H5N1)-like genes in poultry. J Virol 76:507–516. doi:10.1128/jvi.76.2.507-516.2002 11752141 PMC136834

[11] Huang K , Bahl J , Fan XH , Vijaykrishna D , Cheung CL , Webby RJ , Webster RG , Chen H , Smith GJD , Peiris JSM , Guan Y . 2010. Establishment of an H6N2 influenza virus lineage in domestic ducks in Southern China. J Virol 84:6978–6986. doi:10.1128/JVI.00256-10 20463062 PMC2898240

[12] Li J , Quan C , Xie Y , Ke C , Nie Y , Chen Q , Hu T , Chen J , Wong G , Wang Q , Feng L , Yu H , Liu Y , Liu W , Gao GF , Liu WJ , Shi W , Bi Y . 2019. Continued reassortment of avian H6 influenza viruses from Southern China, 2014-2016. Transbound Emerg Dis 66:592–598. doi:10.1111/tbed.13037 30300968

[13] Wang G , Deng G , Shi J , Luo W , Zhang G , Zhang Q , Liu L , Jiang Y , Li C , Sriwilaijaroen N , Hiramatsu H , Suzuki Y , Kawaoka Y , Chen H , Dermody TS . 2014. H6 influenza viruses pose a potential threat to human health. J Virol 88:3953–3964. doi:10.1128/JVI.03292-13 24501418 PMC3993743

[14] Sun H , Kaplan BS , Guan M , Zhang G , Ye J , Long LP , Blackmon S , Yang CK , Chiang MJ , Xie H , Zhao N , Cooley J , Smith DF , Liao M , Cardona C , Li L , Wang GP , Webby R , Wan XF . 2017. Pathogenicity and transmission of a swine influenza A(H6N6) virus. Emerg Microbes Infect 6:e17. doi:10.1038/emi.2017.3 28400591 PMC5457681

[15] Zhao G , Chen C , Huang J , Wang Y , Peng D , Liu X . 2013. Characterisation of one H6N6 influenza virus isolated from swine in China. Res Vet Sci 95:434–436. doi:10.1016/j.rvsc.2013.06.013 23856405

[16] Wei S-H , Yang J-R , Wu H-S , Chang M-C , Lin J-S , Lin C-Y , Liu Y-L , Lo Y-C , Yang C-H , Chuang J-H , Lin M-C , Chung W-C , Liao C-H , Lee M-S , Huang W-T , Chen P-J , Liu M-T , Chang F-Y . 2013. Human infection with avian influenza A H6N1 virus: an epidemiological analysis. Lancet Respir Med 1:771–778. doi:10.1016/S2213-2600(13)70221-2 24461756 PMC7164810

[17] Cheng K , Yu Z , Chai H , Sun W , Xin Y , Zhang Q , Huang J , Zhang K , Li X , Yang S , Wang T , Zheng X , Wang H , Qin C , Qian J , Chen H , Hua Y , Gao Y , Xia X . 2014. PB2-E627K and PA-T97I substitutions enhance polymerase activity and confer a virulent phenotype to an H6N1 avian influenza virus in mice. Virology 468–470:207–213. doi:10.1016/j.virol.2014.08.010 25194918

[18] Wan Z , Gong J , Sang J , Jiang W , Zhao Z , Lian M , Tang T , Li Y , Kan Q , Xie Q , Li T , Shao H , Gao W , Qin A , Ye J . 2022. Mouse adaptation of H6 avian influenza viruses and their molecular characteristics. Front Microbiol 13:1049979. doi:10.3389/fmicb.2022.1049979 36466692 PMC9713515

[19] de Graaf M , Fouchier RAM . 2014. Role of receptor binding specificity in influenza A virus transmission and pathogenesis. EMBO J 33:823–841. doi:10.1002/embj.201387442 24668228 PMC4194109

[20] Sicca F , Neppelenbroek S , Huckriede A . 2018. Effector mechanisms of influenza-specific antibodies: neutralization and beyond. Expert Rev Vaccines 17:785–795. doi:10.1080/14760584.2018.1516553 30145912

[21] Gerhard W , Yewdell J , Frankel ME , Webster R . 1981. Antigenic structure of influenza virus haemagglutinin defined by hybridoma antibodies. Nature 290:713–717. doi:10.1038/290713a0 6163993

[22] Caton AJ , Brownlee GG , Yewdell JW , Gerhard W . 1982. The antigenic structure of the influenza virus A/PR/8/34 hemagglutinin (H1 subtype). Cell 31:417–427. doi:10.1016/0092-8674(82)90135-0 6186384

[23] Smith DJ , Lapedes AS , de Jong JC , Bestebroer TM , Rimmelzwaan GF , Osterhaus ADME , Fouchier RAM . 2004. Mapping the antigenic and genetic evolution of influenza virus. Science 305:371–376. doi:10.1126/science.1097211 15218094

[24] Carnaccini S , Perez DR . 2020. H9 influenza viruses: an emerging challenge. Cold Spring Harb Perspect Med 10:a038588. doi:10.1101/cshperspect.a038588 31871234 PMC7263090

[25] Wan Z , Kan Q , Zhao Z , Shao H , Deliberto TJ , Wan X-F , Qin A , Ye J . 2021. Characterization of subtype H6 avian influenza A viruses isolated from wild birds in Poyang lake, China. Front Vet Sci 8:685399. doi:10.3389/fvets.2021.685399 34589532 PMC8473872

[26] Wei Y , Xu G , Zhang G , Wen C , Anwar F , Wang S , Lemmon G , Wang J , Carter R , Wang M , Sun H , Sun Y , Zhao J , Wu G , Webster RG , Liu J , Pu J . 2016. Antigenic evolution of H9N2 chicken influenza viruses isolated in China during 2009-2013 and selection of a candidate vaccine strain with broad cross-reactivity. Vet Microbiol 182:1–7. doi:10.1016/j.vetmic.2015.10.031 26711021 PMC5029119

[27] Pu J , Wang S , Yin Y , Zhang G , Carter RA , Wang J , Xu G , Sun H , Wang M , Wen C , Wei Y , Wang D , Zhu B , Lemmon G , Jiao Y , Duan S , Wang Q , Du Q , Sun M , Bao J , Sun Y , Zhao J , Zhang H , Wu G , Liu J , Webster RG . 2015. Evolution of the H9N2 influenza genotype that facilitated the genesis of the novel H7N9 virus. Proc Natl Acad Sci U S A 112:548–553. doi:10.1073/pnas.1422456112 25548189 PMC4299237

[28] Krammer F . 2015. Emerging influenza viruses and the prospect of a universal influenza virus vaccine. Biotechnol J 10:690–701. doi:10.1002/biot.201400393 25728134

[29] Krilov LR . 2002. Palivizumab in the prevention of respiratory syncytial virus disease. Expert Opin Biol Ther 2:763–769. doi:10.1517/14712598.2.7.763 12387675

[30] Mulangu S , Dodd LE , Davey RT , Tshiani Mbaya O , Proschan M , Mukadi D , Lusakibanza Manzo M , Nzolo D , Tshomba Oloma A , Ibanda A , et al. . 2019. A randomized, controlled trial of ebola virus disease therapeutics. N Engl J Med 381:2293–2303. doi:10.1056/NEJMoa1910993 31774950 PMC10680050

[31] O’Brien MP , Forleo-Neto E , Musser BJ , Isa F , Chan K-C , Sarkar N , Bar KJ , Barnabas RV , Barouch DH , Cohen MS , et al. . 2021. Subcutaneous REGEN-CoV antibody combination to prevent COVID-19. N Engl J Med 385:1184–1195. doi:10.1056/NEJMoa2109682 34347950 PMC8362593

[32] Köhler G , Milstein C . 1976. Derivation of specific antibody-producing tissue culture and tumor lines by cell fusion. Eur J Immunol 6:511–519. doi:10.1002/eji.1830060713 825377

[33] Lambré CR , Terzidis H , Greffard A , Webster RG . 1990. Measurement of anti-influenza neuraminidase antibody using a peroxidase-linked lectin and microtitre plates coated with natural substrates. J Immunol Methods 135:49–57. doi:10.1016/0022-1759(90)90255-t 1703190

[34] Wan Z , Ye J , Xu L , Shao H , Jin W , Qian K , Wan H , Qin A . 2014. Antigenic mapping of the hemagglutinin of an H9N2 avian influenza virus reveals novel critical amino acid positions in antigenic sites. J Virol 88:3898–3901. doi:10.1128/JVI.03440-13 24429369 PMC3993533

[35] Shao H , Fan Z , Wan Z , Tian X , Chen H , Perez DR , Qin A , Ye J . 2015. An efficient and rapid influenza gene cloning strategy for reverse genetics system. J Virol Methods 222:91–94. doi:10.1016/j.jviromet.2015.06.001 26057220

